# DNA Barcoding for Species Assignment: The Case of Mediterranean Marine Fishes

**DOI:** 10.1371/journal.pone.0106135

**Published:** 2014-09-15

**Authors:** Monica Landi, Mark Dimech, Marco Arculeo, Girolama Biondo, Rogelia Martins, Miguel Carneiro, Gary Robert Carvalho, Sabrina Lo Brutto, Filipe O. Costa

**Affiliations:** 1 Centre of Molecular and Environmental Biology (CBMA), Department of Biology, University of Minho, Braga, Portugal; 2 Malta Centre for Fisheries Science (MCFS), Fort San Lucjan Marsaxlokk, Malta; 3 Dipartimento di Scienze e Tecnologie Biologiche, Chimiche e Farmaceutiche (STEBICEF), University of Palermo, Palermo, Italy; 4 Modelling and Management Fishery Resources Division (DIV-RP), Instituto Português do Mar e da Atmosfera, Lisboa, Portugal; 5 Molecular Ecology and Fisheries Genetics Laboratory, School of Biological Sciences, Bangor University, Bangor, United Kingdom; University of Calgary, Canada

## Abstract

**Background:**

DNA barcoding enhances the prospects for species-level identifications globally using a standardized and authenticated DNA-based approach. Reference libraries comprising validated DNA barcodes (COI) constitute robust datasets for testing query sequences, providing considerable utility to identify marine fish and other organisms. Here we test the feasibility of using DNA barcoding to assign species to tissue samples from fish collected in the central Mediterranean Sea, a major contributor to the European marine ichthyofaunal diversity.

**Methodology/Principal Findings:**

A dataset of 1278 DNA barcodes, representing 218 marine fish species, was used to test the utility of DNA barcodes to assign species from query sequences. We tested query sequences against 1) a reference library of ranked DNA barcodes from the neighbouring North East Atlantic, and 2) the public databases BOLD and GenBank. In the first case, a reference library comprising DNA barcodes with reliability grades for 146 fish species was used as diagnostic dataset to screen 486 query DNA sequences from fish specimens collected in the central basin of the Mediterranean Sea. Of all query sequences suitable for comparisons 98% were unambiguously confirmed through complete match with reference DNA barcodes. In the second case, it was possible to assign species to 83% (BOLD-IDS) and 72% (GenBank) of the sequences from the Mediterranean. Relatively high intraspecific genetic distances were found in 7 species (2.2%–18.74%), most of them of high commercial relevance, suggesting possible cryptic species.

**Conclusion/Significance:**

We emphasize the discriminatory power of COI barcodes and their application to cases requiring species level resolution starting from query sequences. Results highlight the value of public reference libraries of reliability grade-annotated DNA barcodes, to identify species from different geographical origins. The ability to assign species with high precision from DNA samples of disparate quality and origin has major utility in several fields, from fisheries and conservation programs to control of fish products authenticity.

## Introduction

The Mediterranean Sea is a semi-enclosed basin that embraces the marine area from the North East Atlantic Ocean, at West, to the Aegean Sea, at East. The confluences of marine ichthyofauna migrating from the Atlantic Ocean through the Strait of Gibraltar, from the Red Sea and the Indian Ocean through the Suez Channel, and from the Sea of Marmara and Black Sea through the Dardanelles, depicts a picture of the Mediterranean marine biodiversity characterized by a high species richness and peculiarities, including tropical species as well as endemisms [1], [2], [3].

Hosting 7% of the global marine ichthyofauna [4] the Mediterranean Sea is a fascinating prosperous biodiversity hotspot [5], [6] that captured the interest of numerous marine scientists since ancient times (e.g. Aristoteles) [1].

Holding such richness, the Mediterranean Sea can be elected as a very important scientific cradle in marine sciences. The considerable natural variation driven by distinctive regional evolutionary histories and dynamic anthropogenic pressures [7] presents major challenges in local biodiversity monitoring programmes.

Over the past decade, DNA barcoding has played a facilitatory role for accurate identification of marine ichthyiofauna, thanks to the integration of molecular and traditional taxonomic methods [8]. Such DNA-based method provides a robust and standardized approach for marine species identification, as witnessed by the remarkable boost of species identified [9], as well as its use for various applications [10], as for example fisheries and conservation programs [11]. DNA barcoding has been adopted in numerous studies illustrating its speed, reliability and accessibility [12], [13].

The possibility of compiling taxonomic and molecular data into a globally accessible public database (Barcode of Life Data System, BOLD, http://www.barcodinglife.org) [14], [15], comprising taxonomically diverse reference libraries (e.g. Costa et al. [16]), allows usage by a diverse community of scientists and end-users [12]. Such wide-scale adoption enables global comparisons of putative cosmopolitan species [17] facilitating opportunities for comparisons of different marine environments, as well as tackling issues relating to molecular evolution [18]. The availability of a detailed reference library comprising validated DNA barcodes [16] constitutes a robust platform against which to test query sequences, and it represents a valid tool for attributing species to unknown sequences [19].

The DNA barcoding methodology has been applied recently to identify 98 marine species inhabiting the eastern basin of the Mediterranean Sea [20]. Such studies are yet to be extended to marine ichthyofauna populating one of the highest biodiversity richness spots of the Mediterranean, namely the sea around Sicily and Malta.

Here, we present an extensive account of DNA barcodes for Mediterranean fishes based on the mitochondrial *cytochrome c oxidase subunit I* (COI). We used a query dataset composed of 486 specimens identified morphologically from the central basin of the Mediterranean Sea. DNA barcodes generated from these specimens were then screened against the reference dataset of fish from Portugal, as well as against public databases. There were several reasons to choose a reference library from a different location to our target area. First, the marine ichthyofauna of Portugal and of the extension of the Portuguese Continental Shelf is taxonomically well documented [21], and widely characterised using molecular genetic approaches [16], [22]. Specifically, a published reference library for 102 fish species from Portugal, built on COI data was evaluated for taxonomic reliability and attributed to reliability grades [16]. Despite comparing taxa from two differently highly dynamic areas shaped by the confluence of different seas [1], [16], our approach derives from the considerable overlap, especially of exploited species, in the ichthyofauna from Portugal and the central Mediterranean (www.fishbase.org) [23]. The connection between the Mediterranean Sea and the Atlantic Ocean through the Strait of Gibraltar underpins considerable taxonomic similarity, with more than 50 percent of the Mediterranean taxa being of Atlantic origin [24], together with ongoing gene flow in some species [25]. Concomitantly, we examine intra-species population divergence, since similar comparisons have revealed considerable lineage divergence or suggested the occurrence of cryptic species [26]. The universality of the DNA barcodes is, in part, based on the typical low within-species divergence regardless of geographic separation (see Kochzius et al. [27], Ward [28]). The detection of significant divergence among populations is particularly relevant in the present study, given the clear genetic separation previously reported for several fish species across the Atlantic-Mediterranean transition [25]. It also provides empirical scientific support for conservation measures to tackle biodiversity loss and for sustainable exploitation of shared marine fishery resources among southern European countries.

## Materials and Methods

**Figure 1:**
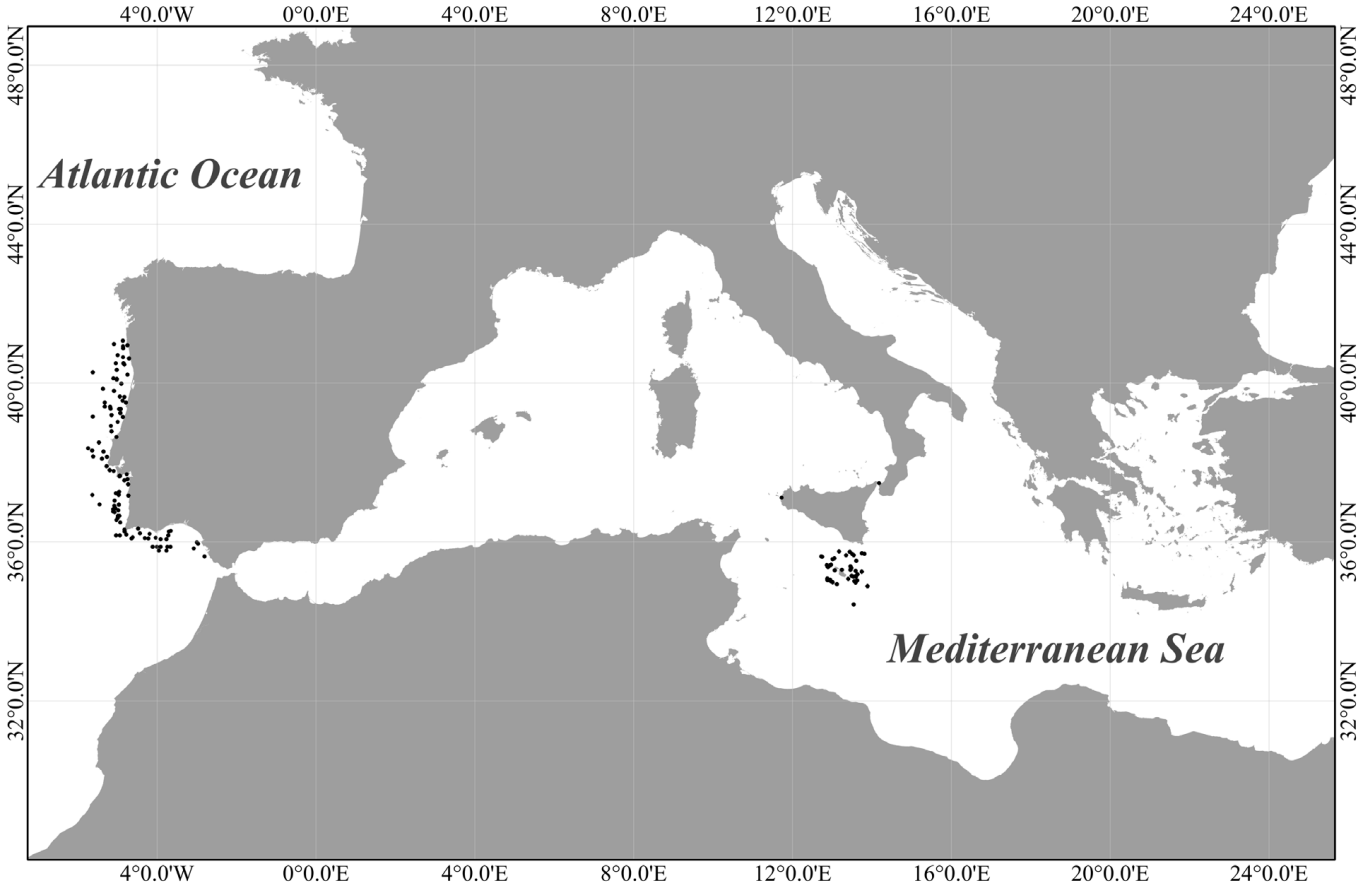
Location of sampling sites. Dots point areas of collection of marine fish specimens along the Portuguese coasts and in the central Mediterranean basin.

In this study we used a reference library of DNA barcodes of fishes from the temperate North East Atlantic, as a core “reference dataset”, to assign species names to a set of fish collected in the central Mediterranean basin, hereafter referred to as “query dataset”. The reference dataset was built using a collection of DNA barcodes of fish from Portugal (Fig. 1), to which taxonomic reliability grades were attributed [16]. The query dataset was composed of DNA barcodes obtained from fish specimens collected in the waters of Malta and Sicily (Italy). In all cases, fish were collected using trawling fishing methods, either on board of research vessels of governmental fisheries research agencies, or directly from legal fisheries landings. No endangered or protected species were sampled. Specific details on specimen collection and DNA barcode generation methods are provided below, separately for the reference and query datasets. Collection and sequencing details for all specimens examined in this study are available in the public project South European Marine Fish: MP (SEFMP), project codes CSFOM, FCFMT, MLFP, lodged in the Barcode of Life Data System (BOLD) [14].

### Preparation of the reference dataset

The reference dataset comprises 792 DNA barcodes from 146 marine fish species collected along the Portuguese continental coast (Fig. 1). 659 DNA barcodes distributed among 102 species of this collection have been previously described, analyzed and verified for their taxonomic reliability through attribution of reliability grades [16]. Five DNA barcodes of the species *Zenion hololepis* have been previously described also [29]. DNA barcodes of the remaining 43 species, and additional 3 genera and 1 family not identified to species level (*n* = 128), collected off Portugal during 2009–2011, were here obtained for the first time, as described in Costa et al. [16]. All DNA sequences generated were characterized by the absence of stop codons, insertions or deletions. Table 1 provides details of the partitioning of the number of species, sequences and GenBank accession numbers among the BOLD projects above mentioned.

**Table 1:** Detailed partitioning of the number of species, COI-5P sequences, and GenBank accession numbers among the BOLD projects examined in this study.

Country	BOLD Project Code	Number of species	Number of COI-5P sequences	Dataset type	Source	GenBank Accessions
Portugal	FCFOP	102	659	Reference	Costa et al. 2012	JQ774505-JQ775163
Portugal	MLFPZ	1	5	Reference	Martins et al. 2012	JF718831-JF718835
Portugal	SEFMP-MLFP	73	128	Reference	Current study	KJ768197-KJ768324
Italy	SEFMP-CSFOM	109	219	Query	Current study	KJ709462-KJ709680
Malta	SEFMP-FCFMT	78	267	Query	Current study	KJ709687-KJ709952

### Preparation of the Mediterranean query dataset

#### Specimen collection

486 specimens, representing 141 marine fish species, were collected from two Mediterranean sub-areas (Fig. 1). 219 specimens were collected from fisheries landings in Sicily, in 2006–2008. 267 samples, analyzed from the Malta area, were collected through the Mediterranean international Bottom trawl Survey (MEDITS), and the Annual national Fisheries data collection program (EC 199/08), in 2006–2007. Specimens were first identified to species level immediately after collection and later verified in the laboratory with the support of taxonomic keys [30], [31], [32], [33]. For each specimen, *c*. 0.5 g of skeletal muscle was dissected with a sterile blade and stored in 96% ethanol.

#### Molecular analyses

Fish tissue samples collected by Malta Centre for Fisheries Science were extracted and amplified as described in Costa et al. [16]. Specimens collected in Sicily were processed at University of Palermo. Total DNA was extracted from the muscle tissue of each specimen, using the DNeasy extraction kit (Qiagen). Sequences of the query dataset were obtained by amplification and sequencing of a 652 bp fragment of 5′ end of the mitochondrial gene *cytochrome c oxidase I* (COI-5P), using the primer pairs FishF1 and FishR1 selected from the primer cocktails described by Ward et al. [17]. Standard PCR reactions were carried out in 12.5 uL total volume, containing about 20 ng of DNA template, 6.25 uL of 10% trehalose, 2 uL of ultrapure water, 1.25 uL of 10X PCR buffer (200 mMTris-HCl pH 8.4, 500 mMKCl), 0.625 uL MgCl2 (50 mM), 0.125 uL of each primer (0.01 mM), 0.0625 uL of each dNTP (10 mM), 0.060 uL of Platinum Taq Polymerase (Invitrogen). The following PCR cycling conditions were employed: 2 min at 95°C; 35 cycles of 0.5 min at 94°C, 0.5 min at 52°C, and 1 min at 72°C; 10 min at 72°C.

PCR products of the query dataset were visualized in a 1% agarose gel and subsequently purified using 10 U of Exonuclease I and 1 U Shrimp Alkaline Phosphatase at 37°C for 15 min, followed by 15 min at 80°C. Both forward and reverse DNA strands were sequenced by using sequencing primers FishF1 or FishR1 [17] and the BigDye Terminator v.3.1 Cycle Sequencing Kit (Applied Biosystems, Inc.) on an ABI 3730 capillary sequencer following manufacturer's instructions.

COI-5P sequences were edited and aligned using MEGA version 5.05 [34], and characterized by the absence of stop codons, insertions or deletions. Sequence data were submitted to BOLD [14], and then deposited in GenBank (http://www.ncbi.nlm.nih.gov), corresponding to accession numbers: KJ709687-KJ709952; KJ709462-KJ709680; KJ768197-KJ768324.

### Data Analyses

#### Reference dataset

The taxonomic reliability of the 128 barcodes, newly added to the reference library of DNA barcodes from the North East Atlantic, was empirically evaluated according to the ranking system described in Costa et al. [16]. The cases of congruence and lack of ambiguities of our barcode data, when compared with specimens retrieved from other public data or projects available in BOLD identification system (BOLD-IDS) [14], conferred a Grade A to that entry. Internal concordance observed only within our dataset, among a minimum of 3 specimens, was equivalent to a Grade B, where the taxonomic reliability decreases due to lack of matching sequences available in BOLD-IDS. Intraspecific sequences with a maximum of 2% (patristic) sequence divergence were attributed to Grade A or Grade B. Intraspecific distances in the COI barcode region have been comprehensively examined in multiple studies involving thousands of marine fish species [35]. These studies consistently revealed that, for the vast majority of the fish species examined (e.g. >98% according to Ward [26] and Knebelsberger et al. [36]), intraspecific distances were <2%. On the other hand, intraspecific distance >2% observed among at least 3 specimens, indicates sub-optimal concordance, and the specimen was then assigned to a Grade C. Lower grade of reliability is expressed by Grades D and E, attributed to sequences represented by a low number of individuals analyzed (1 or 2) and lacking of matching sequence available in BOLD-IDS (Grade D), or sequences with discordant species assignment, from matches with a different species or displaying paraphyly or polyphyly [16].

Pair-wise distances at different taxonomic levels (conspecific, congeneric, and confamilial) were estimated for the reference dataset by using the Kimura 2-parameter (K2P) distance model [37], implemented in BOLD (Distance Summary tool). The analyses were initially run by using the whole dataset of validated sequences, and then repeated by considering only sequences assigned to Grades A and B (689 entries), in order to exclude less reliable entries [16].

#### Molecular identification of species from the query dataset

To assure the blind use of query sequences in molecular assignments, taxonomic identifications initially attributed to the specimens of the query dataset were temporarily removed. First, we used only the reference dataset to build a Neighbour-Joining tree (NJ) based on the K2P distance model and generated using MEGA version 5.05 [34]. Bootstrap values for each branch node were estimated by 1000 replications. Subsequently, we added the query sequences and inspected their position in the tree. Whenever a query sequence occurred within a monophyletic cluster with <2% intra-cluster divergence, it would be assigned to the reference species forming that cluster. Sequences lacking similarities and not matching within existing clusters were representative sequences for a new cluster.

In the case of entries from the query dataset that would not match with reference sequences ranked to grade A [16], additional external confirmation was required. Species assignments were therefore verified by submitting the query sequence to the search engines BOLD Identification System (BOLD-IDS) [14] and GenBank's BLAST [38]. To avoid cross internal verification with our reference dataset, sequences already publicly available from Costa et al. [16], were not considered for matches. Specimen assignment to species was based on a minimum of 98% pair-wise sequence identity over the whole length of the barcode. If more than one matching species was found within the 98–100% identity range, the assignment was made to the one showing the highest identity. Sequences lacking matching clusters or outside the minimum similarity threshold were assumed as first-time COI-5P sequenced species and, therefore, constitute original DNA barcode additions to the global fish barcode library. In those few cases the original species assignment by means of morphological characters was retained, and later confirmed from matching sequences. Finally, we screened the dataset in order to flag species showing intraspecific distances >2% K2P.

## Results

### Reference dataset

The reference dataset comprised COI-5P sequences for 146 species of marine fish (792 specimens), distributed across 115 genera, 70 families, and 25 orders. Eighty-seven species were represented by 3 to 20 individuals per species. Twelve species of the reference dataset constituted new additions to the global library of published COI-5P barcodes for marine fish (Table S1). 122 species, corresponding to 689 DNA barcodes, were assigned to grades A and B.

For the reference dataset, within-species K2P mean distance was 22x lower than average congeneric distance (0.39% and 8.91%, respectively). Average confamilial distance was 15.89% (Table 2A). The maximum intraspecific distance (18.74%) was observed for the species *Scorpaena notata*. Minimum congeneric distance (1.09%) was observed for the genus *Trachurus*. When estimating genetic distances at different taxonomic levels by using the subset of 689 sequences graded as A and B (Table 2B), mean intra-specific distance (0.28%) was 29x lower than mean inter-specific distance (8.38%), while within-family distance was 15.61%. The genus *Microchirus* had the highest within-genus distance (23.06%), while the genus *Trachurus* had the lowest (1.09%).

**Table 2:** Pair-wise COI-5P barcode distances (expressed in %; K2P model) of marine fish species from the reference barcode library, at different taxonomic levels.

A				
Comparison (Intra-)	Comparison (N)	Minimum Distance	Mean Distance± SE	Maximum Distance
**Species**	3452	0	0.39±0	18.74
**Genus**	2019	1.09	8.91±0	23.06
**Family**	4884	3.12	15.89±0	31.28

B				
**Species**	3321	0	0.28±0	3
**Genus**	1905	1.09	8.38±0	23.06
**Family**	4199	3.12	15.61±0	31.28

A. Values calculated using all 792 COI-5P barcodes representing 146 species from the reference library. B. Values calculated using the subset of 689 COI-5P barcodes (122 species) assigned to Grades A and B.

### Query dataset

The query dataset was formed by 141 putative species (based on morphology) belonging to 110 genera, 67 families, and 26 orders, with 1 to 16 specimens per species. Sixty-five species were represented by 3–16 specimens. Seven of the species analysed from the Mediterranean Sea, namely *Aphanius fasciatus* (11 specimens), *Leucoraja melitensis* (1 specimen), *Pomatoschistus tortonesei* (2 specimens), *Raja radula* (1 specimen), *Solea aegyptiaca* (1 specimen), *Squatina aculeata* (1 specimen), and *Tetrapturus belone* (1 specimen), occur exclusively in this area [23]. An additional species *Scorpaenodes arenai* (1 specimen) has a narrow distribution limited to the Azores, in the North East Atlantic, and to the Strait of Messina, in the Mediterranean Sea [23]. Twelve species of the query dataset constitute first-time additions to the public marine fish DNA barcode library.

### Reference and Query datasets – joint analyses

Based on morphological identification, the merged dataset resulted in 218 species distributed over 160 genera and 91 families. Overall, 34.9% of the species (76 species) were collected in the North East Atlantic only, 33.1% (72 species) only from the Mediterranean Sea, and 32.0% (70) were common to both areas (Table S1). To assess the rate of identification success of the molecular assignments here tested, species names were temporarily removed from all query sequences, and subsequently tested against the DNA barcodes of the reference dataset.

### Species identification through molecular assignment

The global NJ tree yielded 233 monophyletic clusters with less than 2% within-cluster divergence (Fig. S1). Sixty-four single-entry clusters were generated, 29 belonging to the reference dataset and 35 to the query dataset. 65% of the query barcodes (*n* = 314) matched with a reference barcode, among which 98% (308 specimens) were unambiguously confirmed through complete match (100% similarity) (Fig. S1). Three clusters (*n* = 6) showed species mismatches (*Diplodus vulgaris*/*D. sargus*, *Epinephelus costae*/*Mycteroperca rubra*, and *Diaphus holti*/*Lobianchia gemellarii*) when the reference cluster assignments were compared with the original morphology-based identifications of the query sequences. Query specimens could not be assigned to species in 35.4% (172 sequences) of the cases, for the lack of corresponding species in the reference dataset. When query sequences were checked for their similarity with homologous sequences in public databases, BOLD-IDS returned 83% (143 entries) of the query sequences as unequivocally identified, by a minimum of 98% similarity with a matching species. 2% (3 entries distributed across 3 species) of the query sequences showed species mismatch, while 15% (26 entries/8 species) had no matching species (Table 3). BLAST searches allowed confirmation of the identity of 72% of the query sequences (*n* = 123), while 8% (14 entries/6 species), matched with a different species, and 20%, equivalent to 35 sequences representing 9 species, had no matching sequences (Table 3).

**Table 3:** Nearest matches (BOLD, GenBank) in the identification of the specimens from the central Mediterranean query dataset.

Species	BOLD	GenBank
***Aphanius fasciatus***	*Aphanius fasciatus* (100%)	*Aphanius anatoliae* (87%)
***Carapus acus***	*Carapus bermudensis* (94%)	*Carapus bermudensis* (94%)
***Diaphus holti***	*Diaphus rafinesquii* (99%)	*Diaphus rafinesquii* (99%)
***Epigonus telescopus***	*Epigonus denticulatus* (100%)*/*	*Epigonus denticulatus* (99%)
	*Howellas herborni* (100%)	
***Epinephelus caninus***	*Mycteroperca rosacea* (93%)	*Epinephelus poecilonotus* (93%)
***Hymenocephalus italicus***	*Hymenocephalus longiceps* (83%)	*Pseudonezumia flagellicauda* (82%)
***Hyporthodus haifensis***	*Epinephelus chabaudi* (98%)	*Epinephelus chabaudi* (98%)
***Labrus merula***	*Labrus merula* (100%)	*Labrus merula* (100%)
***Leucoraja melitensis***	*Leucoraja melitensis* (100%)	*Rajiidae* (99%)
***Mustelus asterias***	*Mustelus asterias* (100%)*/*	*Mustelus palumbes* (99%)/
	*M. palumbes* (100%)/*M. sp.* (100%)	*M. lenticulatus* (99%)
***Mycteroperca rubra***	*Epinephelus costae* (100%)	*Epinephelus costae* (99%)
***Pegusa impar***	*Pegusa lascaris* (95%)	*Pegusa lascaris* (98%)
***Scorpaenodes arenai***	*Scorpaenodes sp. SGP-2010* (88%)	*Scorpaenodes sp. SGP-2010* (88%)
***Syngnathus acus***	*Syngnathus typhle* (96%)	*Symphodus tinca* (99%)
***Tetrapturus belone***	*Tetrapturus belone* (100%)*/*	*Tetrapturus pfluegeri* (100%)
	*T. angustirostris* (100%)*/*	
	*T. pfluegeri* (100%)	
***Trachinus radiates***	*Trachinus radiatus* (100%)	*Trachinus draco* (90%)
***Trisopterus capelanus***	*Trisopterus luscus* (96%)	*Trisopterus luscus* (96%)

Only species that did not match any sequence of the reference dataset are reported.

Values within parenthesis express the percentage of similarity (BOLD) and of identity (GenBank).

### Cases of relatively high intraspecific genetic distances

Nine species showed intraspecific distances greater than 2% K2P and also displayed two well supported sub-specific clusters in the NJ tree (Fig. S1), namely: *Sarda sarda* (2.2%), *Raja montagui* (3.0%), *Coris julis* (3.3%), *Nezumia sclerorhynchus* (4.3%), *Diplodus annularis* (6.0%), *Scorpaena scrofa* (6.3%), *Diplodus sargus* (8.0%), *Spicara maena* (10.9%), and *Scorpaena notata* (18.74%). Moreover, in five of these species (*C. julis*, *D. annularis*, *N. sclerorhyncus*, *S. maena*, and *R. montagui*) the specimens sorted among the two clusters according to their geographic origin, i.e. specimens from NE Atlantic all grouped in one cluster while Mediterranean specimens all grouped in the other.

## Discussion

### DNA barcode-based assignments

We present a test case to the DNA barcode-based identification of 141 putative marine fish species collected in one of the richest marine biodiversity spots of the Mediterranean basin, the Sea around Sicily and Malta. We used a public reference library of DNA barcodes representing 146 marine species from the temperate NE Atlantic to assign species to 486 query sequences from specimens collected in the central Mediterranean. 65% of query sequences matched with the reference barcodes. Successful unambiguous species assignments were obtained in the majority of cases (98%) where matching sequences (and matching species) were available in the reference dataset. Because the reference dataset is composed of a high percentage of species-barcodes annotated with grade A or B (87%), these assignments can be considered very robust.

The successful assignments typically displayed query sequences embedded within the reference haplotypes' cluster, thus showing little or no divergence between North East Atlantic and Mediterranean populations (66 species). These results support our initial premise on the feasibility of using an annotated reference library from the temperate North East Atlantic for species assignments across a neighbouring oceanic basin (i.e. the Mediterranean Sea), and strengthens the robustness of DNA barcode-based approaches for fish species identifications regardless of geographic distance, as observed elsewhere [28], [36], [39].

However, because the overlap of the species analyzed in the two datasets (reference *versus* query) was only partial, a number of sequences could not be assigned using reference library from the North East Atlantic. Nevertheless, through BOLD-IDS and GenBank searches it remained possible to assign species to 83% and 72%, respectively, of these sequences. For both BOLD-IDS and GenBank, however, the reliability of the assignments could not be confirmed or verified due to the presence of unpublished sequences in these databases.

### Mismatches and ambiguities in species assignments

The small percentage of mismatches and ambiguous assignments detected (1.91%) are not necessarily failure of the DNA barcodes to discriminate among species. On the contrary, as discussed further below, most mismatches probably result from species complexes with non-stabilized taxonomic classifications, pending taxonomic revisions and clarifications, morphology-based misidentifications and differential interpretations of the validity of synonyms. A sub-set of the mismatches may have resulted from potential cryptic species. Several studies examining DNA barcodes of the ichthyofauna from other oceanic regions have found similar mismatches and ambiguities between DNA barcode data and current taxonomic knowledge in a low percentage of the species examined [36], [40], [41]. In fact, we did not find any case in our marine fish species dataset with an apparent inability of COI to distinguish species. For example, despite the set of three congeneric species of *Trachurus* in our dataset (*Trachurus picturatus*, *Trachurus mediterraneus* and *Trachurus trachurus*) exhibiting atypically low average congeneric distances (*Trachurus* spp., mean distance 2.28%, minimum distance 1.09%), each species formed an independent branch in the NJ tree, with a unique set of COI-5P haplotypes. Apparently, the low sequence differentiation among species of *Trachurus* is not an exclusive feature of the COI-5P region, and equally low distances have been observed at other locus, namely cytochrome b [42].

Through the analyses of BOLD tools (namely BOLD-IDS generated trees, data not shown), as well as published sequences and literature, we were able to infer the most likely explanation for the observed mismatches and ambiguities. For example, the mismatches involving the pair *Diplodus sargus*/*Diplodus vulgaris* apparently resulted from morphology based-misidentification of the former. DNA barcodes separate clearly these two species, as displayed in the BOLD-IDS tree (data not shown), where each species forms a separate branch with specimens from multiple locations. Hence, in this case, it appears likely that the two specimens from Portugal previously published as *D. sargus* [16] are indeed *D. vulgaris* that match with conspecifics of the query dataset from Sicily.

A number of other mismatches appear to be associated with unstable taxonomic status of some species complexes and their synonyms, as for example with the pairs *Diaphus holti*/*Lobianchia gemellarii*, *Mycteroperca rubra*/*Epinephelus costae* and *Dipturus batis*/*Dipturus oxyrhincus*. Among the latter pair, the species *Dipturus batis* is often confused with the congeneric *D. oxyrhinchus*, despite morphological and colour differences [43]. However, the validity of *D. batis* has not been confirmed in a recent study on *Dipturus* spp. of the North East Atlantic, using morphological and molecular phylogenetic analyses [44]; see also the annotation for *D. batis* in Carneiro et al. [21].

### Cases of relatively high within-species distances

Among several of the species displaying relatively high within-species distances, possible mis-assignments of some specimens to a morphologically close sister species may have occurred. As opposed to mismatches described above, where DNA and morphology pointed to different species, species misidentifications were not obvious. Such discrepancies may derive from a lack of DNA barcode data for all established species within a given group. This could be the case of the small red scorpionfish *Scorpaena notata*, which occurs in two highly divergent branches (18.74%) in the BOLD-IDS tree, neither one matching with any other *Scorpaena* spp., and too divergent to be presumed to belong to the same species. Both branches comprise specimens from different origins, either from North East Atlantic or the Mediterranean. One of the clusters comprises several specimens from continental Portugal, Azores, Italy (Sicily and Liguria), Israel and Mediterranean Spain (Valencia). The other branch includes only 3 specimens, 2 from continental Portugal and 1 from Malta. According to the Food and Agricultural Organization [45], *S. notata* can be easily confused with the cadenat's rockfish *Scorpaena loppei*, and both species occur on the Mediterranean and the Portuguese coast [23], [21]. However, for *S. loppei* there is no sequence data currently available in BOLD nor in GenBank. Furthermore, the whole group is challenging from the viewpoint of identification [46]. Considering only the temperate North East Atlantic, there are 8 additional species reported [21] not included here (*Scorpaena azorica, Scorpaena canariensis, Scorpaena elongata, Scorpaena laevis, Scorpaena maderensis, Scorpaena plumieri, Scorpaena porcus, Scorpaena stephanica*), with only 3 of them having sequence data available in BOLD (data not shown). In the other scorpionfish investigated in our study – the red scorpionfish *Scorpaena scrofa* - we have also found intraspecific structure. Albeit in this case the divergence is not as high as with *S. notata*, it is high (6.3%) compared to variance typical of COI-5P divergence in marine fish [9], and it is likely to reflect the existence of 2 separate species. The clustering pattern for *S. scrofa* patent on the BOLD-IDS tree is somewhat intriguing. Our specimens from Malta (the only location where we collected this species) separated into two branches where they cluster together with specimens from South Africa. Still, a third, less divergent cluster, comprises specimens solely from Turkey [20]. Thus it appears that between 2 to 3 species may be included, where two of them have a wide, and at least partially overlapping, distribution range. Several synonyms are known for this species (e.g. *Scorpeana natalensis*, *Scorpaena lutea* [23]), warranting a taxonomic reconsideration of the genus. Alternatively to the above, high conspecific divergences may reflect actual cases of undescribed species or sub-species. The high divergence detected between North East Atlantic and Mediterranean specimens for the annular seabream *Diplodus annularis* (6.0%) appears to coincide with such hitherto undescribed species diversity. Specimens from this species form a clearly separated branch in the BOLD-IDS tree (data not shown), without any mismatching specimens. Further intraspecific genetic structure is visible in addition to our observations, where between 3 to 5 (or even 6) separate lineages can be discriminated. Notably, the *D. annularis* cluster includes several specimens from Turkey [20] that split in two branches, one closer to the Mediterranean specimens, and the other one to the North East Atlantic. There are DNA barcode sequences available for most *Diplodus* spp. occurring in the Mediterranean, and the only known synonym for *D. annularis* is a case of genus relocation (*Sparus annularis*), therefore reinforcing the case of hidden diversity within this species.

Sympatric populations from Portugal of the Atlantic bonito *Sarda sarda* display moderate COI-5P intraspecific distances. Specimens from Portugal match into two different groups separated by 2.2% genetic distance. There are no other species of *Sarda* spp. recorded for North East Atlantic and Mediterranean, and our sequences do not match any other *Sarda* spp. in BOLD. While we examine only a small sample size, some junior synonyms such as *Sarda mediterranea* (Bloch & Schneider, 1801) would benefit further attention.

Complete sorting between lineages was observed for the species *Nezumia sclerorhynchus*, displaying 7.1% genetic distance between 4 individuals analysed from the North East Atlantic and 2 from Malta, probably reflecting spatial genetic differentiation between the two areas. When verified in BOLD-IDS (data not shown), two clusters were observed. *N. sclerorhyncus* from the North East Atlantic did not match with specimens from other projects. Two *N. sclerorhyncus* specimens collected in Malta matched with conspecifics from Israel. The genetic distance between the two groups (North East Atlantic *versus* Malta) (7.1%) was considerably higher than the 2% observed between the *N. sclerorhynchus* cluster from North East Atlantic and specimens identified as *N. namatahi* from New Zealand.

On first inspection, our findings of high intraspecific distances within the Mediterranean rainbow wrasse *Coris julis* and the blotched picarel *Spicara maena* could be attributed to failure to recognise synonyms as valid species. According to FishBase [23], *C. julis* has a valid sister species *Coris atlantica*, known from the North East Atlantic from Cape Verde archipelago to the coast of Liberia. This species has been confirmed initially by 12S mtDNAs [47] and later using microsatellite analyses [48]. Notably, our specimens from Portugal and the central Mediterranean match closely sequences of *C. atlantica* and *C. julis* respectively published by Kazancioglu et al. [49]. This could indicate that the *C. atlantica* specimens from Portugal were identified incorrectly as *C. julis*. However, the inspection of the BOLD-IDS tree reveals a second and distant branch containing two unpublished sequences of *C. atlantica*, amongst other clusters of *Coris* spp. Adding to these somewhat ambiguous findings, previous mtDNA and microsatellite studies [47], [48] have found some genetic differentiation between Atlantic and Mediterranean populations of *C. julis*, though results with the current work are not directly comparable because a different mtDNA gene was assayed, namely 12 s rDNA. Regarding the above mentioned picarel, our findings point to the existence of two separate species under the specimens identified as *S. maena* (10.9% within-species divergence). According to the BOLD-IDS tree, the specimen from Malta groups with another conspecific from France and two other published sequences [50], while specimens from Portugal, Israel and Italy cluster in a separate branch, where one single specimen assigned to *Spicara flexuosa* from Liguria, Italy, can also be found. There has been some dispute over the validity of *S. flexuosa*. Several sources consider it a junior synonym of *S. maena* [51], including FishBase [23]. Nevertheless, recent 16S rRNA sequence data appear to confirm very distinctly *S. maena* and *S. flexuosa* as two separate species [51]. Therefore, one of the lineages of *S. maena* we have detected could be possibly *S. flexuosa*.

Overall, we report a high level of congruence between current established taxonomic boundaries and the aggregation of DNA barcode sequences in NJ tree's branches, for most species investigated (96%). Still, the detection of several taxonomic discrepancies and of unusual levels of within-species divergence (2.02–18.74%), appear to reflect both the ongoing quest to secure robust and congruent morphology-based identifications in some species complexes, as well as possible overlooked species diversity. Mediterranean ichthyofauna diversity is likely to be especially vulnerable to such challenges. The lack of physical barriers, which have restricted migrations historically, i.e. from the cold North East Atlantic, via the Strait of Gibraltar [25], and from the warm waters of the Red Sea, through the Suez Canal [5], [6], allow greater dispersal and potential gene flow [2]. Furthermore, the complexity of population connectivity obstacles in the Mediterranean Sea, such as that documented between the two major Mediterranean sub-basins (western vs. eastern) probably contributes to high regional genetic differentiation [52], [53], [54].

Examining DNA barcode variation in multiple species across regions has allowed detection of significant population genetic differentiation, as well as highlighting possible cryptic species [28], [39]. Such information on genetic divergence of fish populations is particularly important in the case of economically relevant fish species. Patterns detected here warrant further scrutiny using additional specimens and populations, and detailed examination with complementary morphological and molecular approaches. Misidentifications frequently bias datasets due to various factors, such as lack of taxonomic expertise, operational errors within the DNA barcoding pipeline, as well as morphological ambiguities across different species.

### Concluding Remarks

Here, the molecular identification of the marine ichthyofauna of the central basin of the Mediterranean Sea was verified by screening sequences against an accessible and curated reference library of DNA barcodes. Such a molecular approach, independent from morphology-based taxonomic identification, becomes crucial whenever the input from expert taxonomists is not possible, or when rare or invasive species occur [19]. The ability to identify species starting from tissue of unknown provenance, allows the employment of DNA barcodes in any application where the identification of the whole specimens is not possible. Annotated reference libraries of DNA barcodes provide a robust backbone for a variety of applications, from fish products authentication [55], [56], [57] to biosecurity [58] to the detection of the illegal use of protected or regulated species [59], and fisheries surveillance and management [11], [57]. Such molecular information is likely to be of key importance for improved stock delimitation of shared fishery resources by southern European countries. Moreover, a sustained analysis of trends in the geographic distribution of within- and among-species divergence will not only further elucidate the environmental and demographic factors impacting on marine biodiversity, but importantly also enable a consideration of threats and responses to ongoing anthropogenic change.

Currently, the main limitation appears to be the relative dearth of species' COI sequences in the reference database [60]. The occurrence of taxonomic ambiguities could be at least partially circumvented by the continuous revision and attribution of reliability grades (e.g. Knebelsberger et al. [36]). The expected expansion of the reference libraries of DNA barcodes for fish, as multiple contributions proceed [35], will likely disclose common taxonomic misidentifications and ambiguities, therefore providing opportunities for revision and clarification. The continuous growth of the reference datasets will also enable more among-region comparisons, and assist detection of unusual divergence levels among populations.

## Supporting Information

Figure S1 **Neighbour Joining tree of marine fishes from temperate North East Atlantic (NEA) and Mediterranean Sea (MED).** NJ Tree resulting from 1278 sequences and obtained using Kimura-2-parameter distance model. Branches are collapsed at species level and supported by bootstrap values based on 1000 replicates. For each species, the number of specimens analyzed in each region is reported within parenthesis. For congruence with Costa et al. (2012) [16], specimens identified at genus or family level are presented. In total, 218 species, 160 genera, and 91 families are here reported.
(PDF)

Table S1 **List of 218 species analyzed and respective area of collection.** NEA: North East Atlantic; MED: Mediterranean Sea; NEA and MED: species collected from both areas. † denotes the species newly analyzed in the reference dataset, compared to Costa *et al.* [16]. □ new addition to the global COI-5P library. * Species with distribution limited to the Archipelago of the Azores and to the Strait of Messina. ** Species occurring only in the Mediterranean Sea (www.fishbase.org).
(DOC)

## References

[1] Coll M, Piroddi C, Steenbeek J, Kaschner K, Ben RaisLasram F, et al (2010) The biodiversity of the Mediterranean Sea: Estimates, Patterns, and Threats. PLoS ONE 5: e11842. 20689844 10.1371/journal.pone.0011842 PMC2914016

[2] Azzurro E, Moschella P, Maynou F (2011) Tracking Signals of Change in Mediterranean Fish Diversity Based on Local Ecological Knowledge. PLoS ONE 6: e24885. 21966376 10.1371/journal.pone.0024885 PMC3178559

[3] Lo Brutto S, Arculeo M, Grant WS (2011) Climate change and population genetic structure of marine species. Chem Ecol 27: 107–119.

[4] Bianchi CN, Morri C (2000) Marine biodiversity of the Mediterranean Sea: situation, problems and prospects for future research. Mar Pollut Bull 40: 367–376.

[5] FAO (2003a) Trends in oceanic captures and clustering of large marine ecosystems—2 studies based on the FAO capture database. FAO fisheries technical paper 435. 71 p.

[6] FAO (2003b) Fisheries Management-2. The Ecosystem Approach to Fisheries. FAO Technical Guidelines for Responsible Fisheries 4. 112 p.

[7] Coll M, Piroddi C, Albouy C, Ben Rais Lasram F, Cheung WWL, et al (2011) The Mediterranean Sea under siege: spatial overlap between marine biodiversity, cumulative threats and marine reserves. Glob Ecol Biogeogr 21: 465–480 10.1111/j.1466-8238.2011.00697.x

[8] Hebert PDN, Cywinska A, Ball SL, de WaardJR (2003) Biological identification through DNA barcodes. Proc R Soc Lond B 270: 313–322. 10.1098/rspb.2002.2218 PMC1691236 12614582

[9] Ward RD, Hanner R, Hebert PDN (2009) The campaign to DNA barcode all fishes, FISH-BOL. J Fish Biol 74: 329–356. 20735564 10.1111/j.1095-8649.2008.02080.x

[10] Joly S, Davies TJ, Archambault A, Bruneau A, Derry A, et al (2014) Ecology in the age of DNA barcoding: the resource, the promise and the challenges ahead. Mol Ecol Res 14: 221–232. 10.1111/1755-0998.12173 24118947

[11] Costa FO, Carvalho GR (2007) The Barcode of Life Initiative: synopsis and prospective societal impacts of DNA barcoding of fish. Genomics, Society and Policy 3: 29–40.

[12] Costa FO, Antunes PM (2012) The contribution of the Barcode of Life initiative to the discovery and monitoring of Biodiversity. In: Mendonça, A., et al. (Ed.). Natural Resources, Sustainability and Humanity - A Comprehensive View. pp. 37–68.

[13] Handy SM, Deeds JR, Ivanova NV, Hebert PD, Hanner RH, et al (2011) A single-laboratory validated method for the generation of DNA barcodes for the identification of fish for regulatory compliance. J AOAC Int 94: 201–210. 21391497

[14] Ratnasingham S, Hebert PDN (2007) BOLD: The Barcode of Life Data System (www.barcodinglife.org). Mol Ecol Notes. 7: 355–364. 10.1111/j.1471-8286.2007.01678.x PMC1890991 18784790

[15] Steinke D, Hanner R (2010) The FISH-BOL collaborators' protocol. Mitochondrial DNA 21: 1–5. 10.3109/19401736.2010.536538 21261495

[16] Costa FO, Landi M, Martins R, Costa MH, Costa ME, et al (2012) A ranking system for reference libraries of DNA barcodes: application to marine fish species from Portugal. PLoS ONE 7: e35858. 22558244 10.1371/journal.pone.0035858 PMC3338485

[17] Ward RD, Zemlak TS, Innes BH, Last PR, Hebert PDN (2005) DNA barcoding Australia's fish species. Philos T Roy Soc B 360: 1847–1857. 10.1098/rstb.2005.1716 PMC1609232 16214743

[18] Costa FO, Carvalho GR (2010) New insights into molecular evolution: prospects from the Barcode of Life Initiative (BOLI). Theor Biosci 129: 149–157. 10.1007/s12064-010-0091-y 20502980

[19] Bergsten J, Bilton DT, Fujisawa T, Elliott M, Monaghan MT, et al (2012) The effect of Geographical Scale of Sampling on DNA Barcoding. Syst Biol 61: 1–19. 22398121 10.1093/sysbio/sys037 PMC3417044

[20] Keskin E, Atar HH (2013) DNA barcoding commercially important fish species of Turkey. Mol Ecol Res 13: 788–797. 10.1111/1755-0998.12120 23668535

[21] Carneiro M, Martins R, Landi M, Costa FO (2014) Updated checklist of marine fishes (Chordata: Craniata) from Portugal and the proposed extension of the Portuguese continental shelf. Eur J Taxon 73: 1–73.

[22] Lago FC, Vieites JM, Espiñeira M (2012) Development of a FINS- based method for the identification of skates species of commercial interest. Food Control 24: 38–43.

[23] Frose R, Pauly D (2013) FishBase. World Wide Web electronic publication. Available: http://www.fishbase.org. Version (04/2013).

[24] Plan Bleu (2009) UNEP/MAP-Plan Bleu: State of the Environment and Development in the Mediterranean. UNEP/MAP-Plan Bleu, Athens, www.panbleu.org

[25] Patarnello T, Volckaert FA, Castilho R (2007) Pillars of Hercules: is the Atlantic–Mediterranean transition a phylogeographical break? Mol Ecol 16: 4426–4444. 17908222 10.1111/j.1365-294X.2007.03477.x

[26] Ward RD (2009) DNA barcode divergence among species and genera of birds and fishes. Mol Ecol Resour 9: 1077–1085. 21564845 10.1111/j.1755-0998.2009.02541.x

[27] Kochzius M, Seidel C, Antoniou A, Botla SK, Campo D, et al (2010) Identifying Fishes through DNA Barcodes and Microarrays. PLoS ONE 5: e12620. 20838643 10.1371/journal.pone.0012620 PMC2935389

[28] Ward RD, Costa FO, Holmes BH, Steinke D (2008) DNA barcoding shared fish species from the North Atlantic and Australasia: minimal divergence for most taxa, but *Zeus faber* and *Lepidopus caudatus* each probably constitute two species. Aquat Biol 3: 71–78.

[29] Martins R, Costa FO, Murta A, Carneiro M, Landi M (2012) First record of *Zenion hololepis* (Zenionidae) in Portuguese continental waters: the northernmost occurrence in the eastern Atlantic. Mar Biodivers Rec 5: e30.

[30] Tortonese E (1956) Fauna d'Italia (Vol II). Bologna: Calderini. 334 p.

[31] Tortonese E (1970) Fauna d'Italia (Vol X). Bologna: Calderini. 551 p.

[32] Tortonese E (1975) Fauna d'Italia (Vol XI). Bologna: Calderini. 636 p.

[33] Whitehead PJP, Bauchot ML, Hureau JC, Nielsen J, Tortonese E (1984–86) Fishes of the North-eastern Atlantic and the Mediterranean. UNESCO, Paris. 1473 p.

[34] Tamura K, Peterson D, Peterson N, Stecher G, Nei M, et al (2011) MEGA5: Molecular Evolutionary Genetics Analysis using Maximum Likelihood, Evolutionary Distance, and Maximum Parsimony Methods. Mol Biol Evol 28: 2731–2739. 21546353 10.1093/molbev/msr121 PMC3203626

[35] Ward RD (2012) FISH-BOL, A case study for DNA Barcodes. In: Kress JW, Erickson DL, editors. DNA Barcodes Methods and Protocols. pp. 423–439. 10.1007/978-1-61779-591-6_21 22684969

[36] Knebelsberger T, Landi M, Neumann H, Kloppmann M, Sell AF, et al.. (2014) A reliable DNA barcode reference library for the identification of the North European shelf fish fauna. Mol Ecol Res, online in advance of print. doi:10.1111/1755-0998.12238. 10.1111/1755-0998.12238 24618145

[37] Kimura M (1980) A simple method of estimating evolutionary rate of base substitutions through comparative studies of nucleotide sequences. J Mol Evol 16: 111–120. 7463489 10.1007/BF01731581

[38] Altschul SF, Gish W, Miller W, Myers EW, Lipman DJ (1990) Basic local alignment search tool. J Mol Biol 215: 403–410. 2231712 10.1016/S0022-2836(05)80360-2

[39] Zemlak TS, Ward RD, Connell AD, Holmes BH, Hebert PDN (2009) DNA barcoding reveals overlooked marine fishes, Mol Ecol Res. 9: 237–242. 10.1111/j.1755-0998.2009.02649.x 21564983

[40] Mat Jaafar TM, Taylor MI, Nor SAM, Bruyn M, Carvalho GR (2012) DNA barcoding reveals cryptic diversity within commercially exploited Indo-Malay Carangidae (Teleosteii: Perciformes). PLoS ONE 7(11): e49623 10.1371/journal.pone.0049623 23209586 PMC3510217

[41] McCusker MR, Denti D, Van Guelpen L, Kenchington E, Bentzen P (2013) Barcoding Atlantic Canada's commonly encountered marine fishes. Mol Ecol Res 13: 177–188. 10.1111/1755-0998.12043 23253798

[42] Cárdenas L, Hernández CE, Poulina E, Magoulas A, Kornfield I, et al (2005) Origin, diversification, and historical biogeography of the genus *Trachurus* (Perciformes: Carangidae). Mol Phylogenet Evol 35: 496–507. 15804418 10.1016/j.ympev.2005.01.011

[43] Cannas R, Follesa MC, Cabiddu S, Porcu C, Salvadori S, et al (2012) Molecular and morphological evidence of the occurrence of the Norwegian skate *Dipturus nidarosiensis* (Storm, 1881) in the Mediterranean Sea. Mar Biol Res 6: 341–350.

[44] Iglésias SP, Toulhoat L, Sellos DY (2009) Taxonomic confusion and market mislabelling of threatened skates: Important consequences for their conservation status. Aquat Conserv 20: 319–333.

[45] Fischer W, Schneider M, Bauchot ML (1987) Fiches FAO d'identification des espèces pour les besoins de la pêche. FAO, Rome 2: 761–1530.

[46] Ordines F, Valls M, Gouraguine A (2012) Biology, Feeding, and Habitat preferences of cadenat's rockfish, *Scorpaena loppei* (Actinopterygii: Scorpaeniformes: Scorpaenidae), in the Balearic Island (Western Mediterranean). Acta Ichthyologica et Piscatoria 42: 21–30.

[47] Guillemaud T, Cancela ML, Afonso P, Morato T, Santos RS, et al (2000) Molecular insights into the taxonomic status of *Coris atlantica* (Pisces: Labridae). J Mar Biol Assoc U.K. 80: 929–933.

[48] Aurelle D, Guillemaud T, Afonso P, Morato T, Wirtz P, et al (2003) Genetic study of *Coris julis* (Osteichtyes, Perciformes, Labridae) evolutionary history and dispersal abilities. C R Biol 326: 771–785. 14608697 10.1016/j.crvi.2003.08.001

[49] Kazancioglu E, Near TJ, Hanel R, Wainwright PC (2009) Influence of sexual selection and feeding functional morphology on diversification rate of parrot fishes (Scaridae). Proc. R. Soc. B 276: 3439–3446. 10.1098/rspb.2009.0876 PMC2817191 19586949

[50] Yamanoue Y, Miya M, Matsuura K, Yagishita N, Mabuchi K, et al (2007) Phylogenetic position of tetraodontiform fishes within the higher teleosts: Bayesian inferences based on 44 whole mitochondrial genome sequences. Mol Phylogenet Evol 45: 89–101. 17490896 10.1016/j.ympev.2007.03.008

[51] Imsiridou A, Minos G, Gakopoulou A, Katsares V, Karids T, et al (2011) Discrimination of two picarel species *Spicara flexuosa* and *Spicara maena* (Pisces: Centracanthidae) based on mitochondrial DNA sequences. J Fish Biol 78: 373–377. 21235569 10.1111/j.1095-8649.2010.02858.x

[52] Maggio T, Lo Brutto S, Garoia F, Tinti F, Arculeo M (2009) Microsatellite analysis of red mullet *Mullus barbatus* (Perciformes, Mullidae) reveals the isolation of the Adriatic Basin in the Mediterranean Sea. ICES J Mar Sci 66: 1883–1891.

[53] Mejri R, Arculeo M, Hassine B, Lo Brutto S (2011) Genetic architecture of the marbled goby *Pomatoschistus marmoratus* (Perciformes, Gobiidae) in the Mediterranean Sea. Mol Phylogenet Evol 58: 395–403. 21167289 10.1016/j.ympev.2010.12.001

[54] Mejri R, Lo Brutto S, Hassine B, Arculeo M (2009) A study on *Pomatoschistus tortonesei* Miller 1968 (Perciformes, Gobiidae) reveals the Siculo-Tunisian Strait (STS) as a breakpoint to gene flow in the Mediterranean basin. Mol Phylogenet Evol 53: 596–601. 19422922 10.1016/j.ympev.2009.04.018

[55] Barbuto M, Galimberti A, Ferri E, Labra M, Malandra R, et al (2009) DNA barcoding reveals fraudulent substitutions in shark seafood products: The Italian case of ‘‘palombo” (Mustelus spp.). Food Res Int 43: 376–381.

[56] Hajibabaei M, Smith MA, Janzen DH, Rodriguez JJ, Whitfield JB, et al (2006) A minimalist barcode can identify a specimen whose DNA is degraded. Mol Ecol Notes 6: 959–964.

[57] Hanner R, Becker S, Ivanova NV, Steinke D (2011) FISH-BOL and seafood identification: Geographically dispersed case studies reveal systemic market substitution across Canada. Mitochondrial DNA 22: 106–122. 21980986 10.3109/19401736.2011.588217

[58] Armstrong KF, Ball SL (2005) DNA barcodes for biosecurity: invasive species identification. Philos T Roy Soc B 360: 1813–1823. 10.1098/rstb.2005.1713 PMC1609225 16214740

[59] Rasmussen RS, Morrissey MT (2008) DNA-Based Methods for the Identification of Commercial Fish and Seafood Species. Compr Rev Food Sci Food Saf 7: 280–295. 10.1111/j.1541-4337.2008.00046.x 33467804

[60] Cerutti-Pereyra F, Meekan MG, Wei N-WV, O'Shea O, Bradshaw CJA, et al (2012) Identification of Rays through DNA Barcoding: An Application for Ecologists. PLoS ONE 7: e36479 10.1371/journal.pone.0036479 22701556 PMC3372520

