# Supplementary figures and images for: DNA Barcoding for Species Assignment: The Case of Mediterranean Marine Fishes
Source: PLoS One. 2014 Sep 15;9(9):e106135. doi: 10.1371/journal.pone.0106135 (PMC4164363; doi:10.1371/journal.pone.0106135)

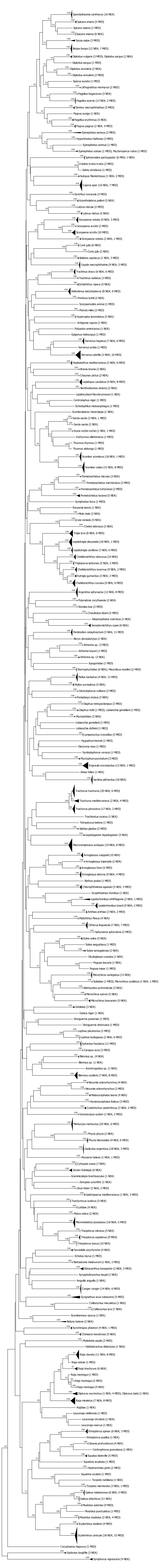

0.05

Supplement: Figure S1 — Neighbour Joining tree of marine fishes from temperate North East Atlantic (NEA) and Mediterranean Sea (MED). NJ Tree resulting from 1278 sequences and obtained using Kimura-2-parameter distance model. Branches are collapsed at species level and supported by bootstrap values based on 1000 replicates. For each species, the number of specimens analyzed in each region is reported within parenthesis. For congruence with Costa et al. (2012) [16], specimens identified at genus or family level are presented. In total, 218 species, 160 genera, and 91 families are here reported. (PDF) [file pone.0106135.s001.pdf]
