# Supplementary material for: DNA Barcoding for Species Assignment: The Case of Mediterranean Marine Fishes
Source: PLoS One. 2014 Sep 15;9(9):e106135. doi: 10.1371/journal.pone.0106135 (PMC4164363; doi:10.1371/journal.pone.0106135)
Supplement: Table S1 — List of 218 species analyzed and respective area of collection. NEA: North East Atlantic; MED: Mediterranean Sea; NEA and MED: species collected from both areas. † denotes the species newly analyzed in the reference dataset, compared to Costa et al. [16]. □ new addition to the global COI-5P library. * Species with distribution limited to the Archipelago of the Azores and to the Strait of Messina. ** Species occurring only in the Mediterranean Sea (www.fishbase.org). (DOC) [file pone.0106135.s002.doc]

**Table S1. List of 218 species, representing 161 genera, and 91 families, analyzed and respective area of collection.**

| **Species** | **NEA** | **MED** | **GenBank A.N.** |
| --- | --- | --- | --- |
| **Family Chimaeridae** |  |  |  |
| *Chimaera monstrosa* | 5 |  | JQ774509;  JQ774596-JQ774599 |
| **Family Scyliorhinidae** |  |  |  |
| *Galeus atlanticus* | 11 |  | JQ774627-JQ774837 |
| *Galeus melastomus* | 6 | 5 | JQ774633-  JQ774634;  JQ774838-  JQ774841; KJ709765-KJ709769 |
| *Scyliorhinus canicula* | 18 | 15 (7^◊^) | JQ774728-  JQ774732;  JQ774911-  JQ774915;  JQ775119-  JQ775126; KJ709891-KJ709898; KJ709614-KJ709620 |
| *Scyliorhinus stellaris* |  | 4 (2^◊^) | KJ709899-KJ709900; KJ709621-KJ709622 |
| **Family Triakidae** |  |  |  |
| *Mustelus asterias* |  | 4 | KJ709829-KJ709832 |
| *Mustelus mustelus* † □ | 2 | 4 | KJ709833-KJ709836; KJ768265-KJ768266 |
| *Mustelus punctulatus* |  | 1 | KJ709837 |
| **Family Hexanchidae** |  |  |  |
| *Heptranchias perlo* |  | 2 | KJ709775-KJ709776 |
| **Family Squalidae** |  |  |  |
| *Squalus blainville* |  | 5 (1^◊^) | KJ709923-KJ709926; KJ709641 |
| **Family Centrophoridae** |  |  |  |
| *Centrophorus granulosus* | 1 |  | JQ774505 |
| *Deania profundorum* | 4 |  | JQ774525-  JQ774528 |
| **Family Etmopteridae** |  |  |  |
| *Etmopterus pusillus* | 1 |  | JQ774531 |
| *Etmopterus spinax* | 6 | 3 | JQ774532;  JQ774612-  JQ774616; KJ709757-KJ709759 |
| **Family Squatinidae** |  |  |  |
| *Squatina aculeata********* |  | 1^◊^ | KJ709642 |
| **Family Torpedinidae** |  |  |  |
| *Torpedo marmorata* | 3 | 1^◊^ | JQ774752-  JQ774753; KJ709652; KJ768313 |
| *Torpedo nobiliana* | 1 |  | JQ774557 |
| *Torpedo torpedo* |  | 1^◊^ | **KJ709653** |
| **Family Rajidae** |  |  |  |
| *Dipturus batis* | 1 |  | JQ774529 |
| *Dipturus oxyrinchus* | 1 | 4 (1^◊^) | JQ774530; KJ709752-KJ709754; KJ709522 |
| *Leucoraja circularis* | 1 |  | JQ774537 |
| *Leucoraja melitensis********* |  | 1^◊^ | KJ709547 |
| *Leucoraja naevus* | 1 |  | JQ774853 |
| *Raja brachyura* | 6 |  | JQ774887;  JQ775097-  JQ775098; KJ768288-KJ768290 |
| *Raja clavata* | 11 | 8 (1^◊^) | JQ774697-JQ774700;  JQ774888-JQ774891;  JQ775099-  JQ775101; KJ709861-KJ709867; KJ709589 |
| *Raja miraletus* | 7 | 8 (3^◊^) | JQ774701-  JQ774704;  JQ774892-  JQ774894; KJ709868-KJ709872; KJ709590-KJ709592 |
| *Raja montagui* | 4 |  | JQ774705-  JQ774707;  JQ774895 |
| *Raja polystigma* |  | 3^◊^ | KJ709593-KJ709595 |
| *Raja radula********* |  | 1^◊^ | KJ709596 |
| **Family Myliobatidae** |  |  |  |
| *Myliobatis aquila* |  | 2^◊^ | KJ709571-KJ709572 |
| **Family Notacanthidae** |  |  |  |
| *Notacanthus bonaparte* † | 2 | 3 | KJ709844-KJ709846; KJ768268-KJ768269 |
| **Family Anguillidae** |  |  |  |
| *Anguila anguilla* † | 1 |  | KJ768203 |
| **Family Muraenidae** |  |  |  |
| *Muraena helena* † □ | 1 | 1 | KJ709828; KJ768264 |
| **Family Synaphobranchidae** |  |  |  |
| *Synaphobranchus kaupii* | 1 |  | JQ774748 |
| **Family Ophichthidae** |  |  |  |
| *Echelus myrus* |  | 1 | KJ709755 |
| **Family Congridae** |  |  |  |
| *Conger conger* | 14 | 6 (1^◊^) | JQ774603-  JQ774607;  JQ774817-  JQ774821;  JQ775004-  JQ775007; KJ709739-KJ709743; KJ709504 |
| **Family Nettastomatidae** |  |  |  |
| *Facciolella oxyrhyncha* | 4 |  | JQ774533-  JQ774535;  JQ774617 |
| *Nettastoma melanurum* † □ | 1 | 2 | KJ709840-KJ709841; KJ768267 |
| **Family Engraulidae** |  |  |  |
| *Engraulis encrasicolus* | 13 | 1^◊^ | JQ774610-  JQ774611;  JQ774823-  JQ774827;  JQ775015-  JQ775020; KJ709524 |
| **Family Clupeidae** |  |  |  |
| *Alosa fallax* † □ | 1 |  | KJ768202 |
| *Sardina pilchardus* | 18 |  | JQ774708-  JQ774712;  JQ774896-  JQ774900;  JQ775102-  JQ775107; KJ768296-KJ768297 |
| **Family Argentinidae** |  |  |  |
| *Argentina sphyraena* | 12 | 8 (1^◊^) | JQ774570-  JQ774571;  JQ774770-  JQ774773;  JQ774960-  JQ774964;  KJ709687-KJ709693; KJ709476; KJ768206 |
| **Family Alepocephalidae** |  |  |  |
| *Alepocephalus rostratus* † □ | 1 |  | KJ768201 |
| *Xenodermichthys copei* | 6 |  | JQ774563-  JQ774568 |
| **Family Salmonidae** |  |  |  |
| *Salmo ohridanus* |  | 1^◊^ | KJ709597 |
| *Salmo trutta trutta* |  | 3^◊^ | KJ709598-KJ709600 |
| **Family Sternoptychidae** |  |  |  |
| *Maurolicus muelleri* |  | 2^◊^ | KJ709557-KJ709558 |
| **Family Phosichthyidae** |  |  |  |
| *Polymetme corythaeola* | 2 |  | JQ774548-  JQ774549 |
| *Vinciguerria attenuata* |  | 1^◊^ | KJ709674 |
| *Vinciguerria poweriae* |  | 1^◊^ | KJ709675 |
| **Family Stomiidae** |  |  |  |
| *Chauliodus sloani* |  | 2 (1^◊^) | KJ709727; KJ709495 |
| *Stomias boa* |  | 2 | KJ709927-KJ709928 |
| **Family Aulopidae** |  |  |  |
| *Aulopus filamentosus* † | 1 | 2^◊^ | KJ709481-KJ709482; KJ768210 |
| **Family Chlorophthalmidae** |  |  |  |
| *Chlorophthalmus agassizi* | 5 | 3 (1^◊^) | JQ774510-  JQ774514; KJ709733; KJ709499 |
| **Family Myctophidae** |  |  |  |
| *Diaphus holti* |  | 1^◊^ | KJ709514 |
| *Diaphus metopoclampus* |  | 3 (1^◊^) | KJ709749-KJ709750; KJ709515 |
| *Electrona risso* |  | 1^◊^ | KJ709523 |
| *Hygophum benoiti* |  | 1^◊^ | KJ709536 |
| *Lampanyctus crocodilus* |  | 5 | KJ709784-KJ709788 |
| *Lobianchia dofleini* |  | 1^◊^ | KJ709550 |
| *Lobianchia gemellarii* |  | 1 | KJ709802 |
| *Myctophum punctatum* |  | 3 (1^◊^) | KJ709838-KJ709839; KJ709570 |
| *Symbolophorus veranyi* |  | 1^◊^ | KJ709643 |
| **Family Macrouridae** |  |  |  |
| *Coelorinchus caelorhincus* | 3 | 2^◊^ | JQ774515-  JQ774517; KJ709502-KJ709503 |
| *Gadomus longifilis* † □ | 3 |  | KJ768230-KJ768232 |
| *Hymenocephalus italicus* |  | 3 | KJ709781-KJ709783 |
| *Malacocephalus laevis* | 4 |  | JQ774538-  JQ774539; KJ768255-KJ768256 |
| *Nezumia sclerorhynchus* | 4 | 2 | JQ774541-  JQ774544; KJ709842-KJ709843 |
| *Trachyrincus scabrus* | 5 |  | JQ774558-  JQ774562 |
| **Family Merlucciidae** |  |  |  |
| *Merluccius merluccius* | 20 | 6 (2^◊^) | JQ774659-  JQ774668;  JQ774859-  JQ774863;  JQ775071-  JQ775075; KJ709813-KJ709816; KJ709559-KJ709560 |
| **Family Phycidae** |  |  |  |
| *Phycis blennoides* | 4 | 6 (1^◊^) | JQ774545-  JQ774547;  JQ774694; KJ709856-KJ709860; KJ709582 |
| *Phycis phycis* † □ | 2 |  | KJ768278-KJ768279 |
| **Family Gadidae** |  |  |  |
| *Gadiculus argenteus* | 18 | 3 (2^◊^) | JQ774618-  JQ774625;  JQ774828-  JQ774832;  JQ775024-  JQ775028; KJ709761; KJ709531-KJ709532 |
| *Gaidropsarus mediterraneus* | 1 | 3 | JQ774626; KJ709762-KJ709764 |
| *Micromesistius poutassou* | 16 | 5 | JQ774682-  JQ774686;  JQ774867-  JQ774871;  JQ775081-  JQ775086; KJ709817-KJ709821 |
| *Molva molva* | 2 |  | JQ774540; KJ768260 |
| *Trisopterus luscus* | 10 |  | JQ774952-  JQ774956;  JQ775154-  JQ775158 |
| *Trisopterus capelanus* |  | 9 (7^◊^) | KJ709948-KJ709949; KJ709666-KJ709672 |
| *Trisopterus minutus* | 5 |  | JQ775159-  JQ775163 |
| **Family Carapidae** |  |  |  |
| *Carapus acus* |  | 3 | KJ709718-KJ709720 |
| **Family Batrachoididae** |  |  |  |
| *Halobatrachus didactylus* | 1 |  |  |
| **Family Lophiidae** |  |  |  |
| *Lophius budegassa* | 2 | 6 (2^◊^) | JQ774651-  JQ774652; KJ709803-KJ709806; KJ709551-KJ709552 |
| *Lophius piscatorius* |  | 2 (1^◊^) | KJ709807; KJ709553 |
| **Family Chaunacidae** |  |  |  |
| *Chaunax pictus* | 2 |  | JQ774506-  JQ774507 |
| **Family Mugilidae** |  |  |  |
| *Chelon labrosus* † | 2 |  | KJ768226-KJ768227 |
| *Liza ramada* | 5 |  | JQ775055-  JQ775059 |
| **Family Atherinidae** |  |  |  |
| *Atherina boyeri* |  | 1^◊^ | KJ709477 |
| **Family Belonidae** |  |  |  |
| *Belone belone* | 2 |  | JQ774572; KJ768216 |
| **Family Scomberesocidae** |  |  |  |
| *Scomberesox saurus* † □ | 1 |  | KJ768299 |
| **Family Cyprinodontidae** |  |  |  |
| *Aphanius fasciatus********* |  | 11^◊^ | KJ709464-KJ709474 |
| **Family Trachichthyidae** |  |  |  |
| *Hoplostethus mediterraneus* † | 2 | 6 (2^◊^) | KJ709777-KJ709780; KJ709534-KJ709535; KJ768238-KJ768239 |
| **Family Berycidae** |  |  |  |
| *Beryx decadactylus* † | 1 |  | KJ768217 |
| **Family Parazenidae** |  |  |  |
| *Cyttopsis rosea* | 7 |  | JQ774518-  JQ774524 |
| **Family Zeniontidae** |  |  |  |
| *Zenion hololepis* † | 6 |  | JF718831-  JF718835; KJ768321 |
| **Family Grammicolepididae** |  |  |  |
| *Grammicolepis brachiusculus* † □ | 1 |  | KJ768237 |
| **Family Zeidae** |  |  |  |
| *Zenopsis conchifer* † □ | 1 |  | KJ768322 |
| *Zeus faber* | 2 | 2 (1^◊^) | KJ709952; KJ709680; KJ768323-KJ768324 |
| **Family Syngnathidae** |  |  |  |
| *Syngnathus acus* |  | 5^◊^ | KJ709646-KJ709650 |
| **Family Macroramphosidae** |  |  |  |
| *Macroramphosus scolopax* | 19 | 8 (3^◊^) | JQ774653-  JQ774658;  JQ774854-  JQ774858;  JQ775064-  JQ775070; KJ709808-KJ709812; KJ709554-KJ709556; KJ768254 |
| **Family Dactylopteridae** |  |  |  |
| *Dactylopterus volitans* |  | 2^◊^ | KJ709511-KJ709512 |
| **Family Scorpaenidae** |  |  |  |
| *Helicolenus dactylopterus* | 8 | 6 (1^◊^) | JQ774635-  JQ774638;  JQ775034-  JQ775038; KJ709770-KJ709774; KJ709533 |
| *Pontinus kuhlii* | 2 |  | JQ774695-JQ774696 |
| *Pterois miles* |  | 2^◊^ | KJ709587-KJ709588 |
| *Scorpaena notata* | 11 | 4 (3^◊^) | JQ774726-  JQ774727;  JQ774906-  JQ774910; KJ709878; KJ709610-KJ709612; KJ768305-KJ768308 |
| *Scorpaena scrofa* |  | 12 | KJ709879-KJ709890 |
| *Scorpaenodes arenai******** |  | 1^◊^ | KJ709613 |
| **Family Triglidae** |  |  |  |
| *Chelidonichthys cuculus* | 9 | 6 (1^◊^) | JQ774974-  JQ774982; KJ709728-KJ709732; KJ709496 |
| *Chelidonichthys lucernus* | 9 | 2^◊^ | JQ774803-  JQ774807;  JQ774998-  JQ775001’ KJ709497-KJ709498 |
| *Chelidonichthys obscurus* | 10 |  | JQ774508;  JQ774594-  JQ774595;  JQ774808-  JQ774812;  JQ775002-  JQ775003 |
| *Eutrigla gurnardus* | 3 | 2 (1^◊^) | JQ775021-  JQ775023; KJ709760; KJ709530 |
| *Lepidotrigla cavillone* | 7 | 6 | JQ774639-  JQ774643;  JQ774848-  JQ774849; KJ709795-KJ709800 |
| *Lepidotrigla dieuzeidei* | 18 | 1 | JQ774644-  JQ774650;  JQ774850-  JQ774852;  JQ775049-  JQ775054; KJ709801; KJ768252-KJ768253 |
| *Trigla lyra* | 8 | 6 (3^◊^) | JQ774767-  JQ774768;  JQ774943-  JQ774946;  JQ775152-  JQ775153; KJ709945-KJ709947; KJ709661-KJ709663 |
| *Trigloporus lastoviza* | 5 | 2^◊^ | JQ774947-  JQ774951; KJ709664-KJ709665 |
| **Family Peristediidae** |  |  |  |
| *Peristedion cataphractum* | 3 | 11 (6^◊^) | JQ774692-  JQ774693; KJ709851-KJ709855; KJ709576-KJ70958; KJ768277 |
| **Family Polyprionidae** |  |  |  |
| *Polyprion americanus* † | 1 |  | KJ768281 |
| **Family Serranidae** |  |  |  |
| *Anthias anthias* | 3 | 2^◊^ | JQ774769;  JQ774959; KJ709462-KJ709463; KJ768204 |
| *Epinephelus aeneus* |  | 2^◊^ | KJ709525-KJ709526 |
| *Epinephelus caninus* |  | 1^◊^ | KJ709527 |
| *Epinephelus costae* |  | 1^◊^ | KJ709528 |
| *Hyporthodus haifensis* |  | 3^◊^ | KJ709537-KJ709539 |
| *Mycteroperca rubra* |  | 1^◊^ | KJ709569 |
| *Serranus cabrilla* | 2 | 16 (8^◊^) | JQ774551; KJ709901-KJ709908; KJ709623-KJ709630; KJ768309 |
| *Serranus hepatus* | 7 | 6 (1^◊^) | JQ774733-  JQ774737;  JQ774916-  JQ774917; KJ709909-KJ709913; KJ709631 |
| *Serranus scriba* |  | 1^◊^ | KJ709632 |
| **Family Epigonidae** |  |  |  |
| *Epigonus telescopus* |  | 1 | KJ709756 |
| **Family Coryphaenidae** |  |  |  |
| *Coryphaena hippurus* |  | 1 | KJ709744 |
| **Family Carangidae** |  |  |  |
| *Trachinotus ovatus* † | 1 |  | KJ768314 |
| *Trachurus mediterraneus* † | 2 | 4^◊^ | KJ709654-KJ709657; KJ768315-KJ768316 |
| *Trachurus picturatus* | 17 | 2^◊^ | JQ774755-  JQ774759;  JQ774933-  JQ774937;  JQ775140-  JQ775146; KJ709658-KJ709659 |
| *Trachurus trachurus* | 20 | 6 (1^◊^) | JQ774760-  JQ774766;  JQ774938-  JQ774942;  JQ775147-  JQ775151; KJ709940-KJ709944; KJ709660; KJ768317-KJ768319 |
| **Family Bramidae** |  |  |  |
| *Brama brama* † | 2 |  | KJ768224-KJ768225 |
| **Family Haemulidae** |  |  |  |
| *Pomadasys incisus* † | 3 |  | KJ768282-KJ768284 |
| **Family Sparidae** |  |  |  |
| *Boops boops* | 12 | 7 (2^◊^) | JQ774575-  JQ774579;  JQ774791-  JQ774793;  JQ774984-  JQ774987; KJ709708-KJ709712; KJ709490-KJ709491 |
| *Dentex macrophthalmus* |  | 5 (1^◊^) | KJ709745-KJ709748; KJ709513 |
| *Diplodus annularis* | 2 | 2^◊^ | JQ774608;  JQ774609; KJ709516-KJ709517 |
| *Diplodus sargus* | 2 | 1^◊^ | JQ774822;  JQ775009; KJ709518 |
| *Diplodus vulgaris* |  | 3^◊^ | KJ709519-KJ709521 |
| *Lithognathus mormyrus* |  | 2^◊^ | KJ709548-KJ709549 |
| *Pagellus acarne* | 12 | 2^◊^ | JQ7746900  JQ774691;  JQ774877-  JQ774881;  JQ775089-  JQ775093; KJ709573-KJ709574 |
| *Pagellus bogaraveo* † | 3 |  | KJ768270-KJ768272 |
| *Pagellus erythrinus* | 5 |  | JQ774882-  JQ774886 |
| *Pagrus auriga* † | 1 |  | KJ768273 |
| *Pagrus pagrus* † | 2 | 4 | KJ709847-KJ709850; KJ768274-KJ768275 |
| *Sarpa salpa* |  | 3^◊^ | KJ709602-KJ709604 |
| *Sparus aurata* |  | 1^◊^ | KJ709635 |
| *Spondyliosoma cantharus* | 16 |  | JQ774743-  JQ774747;  JQ774923-  JQ774927;  JQ775128-  JQ775133 |
| **Family Centracanthidae** |  |  |  |
| *Spicara maena* | 6 | 1 | JQ774738-  JQ774742; KJ709919; KJ768312 |
| *Spicara smaris* |  | 5 (2^◊^) | KJ709920-KJ709922; KJ709639-KJ709640 |
| **Family Mullidae** |  |  |  |
| *Mullus barbatus* | 4 | 13 (7^◊^) | JQ774687; KJ768261-KJ768263 |
| *Mullus surmuletus* | 5 |  | JQ774872-  JQ774876; KJ709822-KJ709827; KJ709562-KJ709568 |
| **Family Cepolidae** |  |  |  |
| *Cepola macrophthalma* | 9 | 5 | JQ774589-  JQ774593;  JQ774994-  JQ774997; KJ709722-KJ709726 |
| **Family Labridae** |  |  |  |
| *Acantholabrus palloni* | 5 |  | JQ774957-  JQ774958; KJ768197-KJ768199 |
| *Coris julis* † | 2 | 6^◊^ | KJ709505-KJ709510; KJ768228-KJ768229 |
| *Labrus merula* |  | 2^◊^ | KJ709540-KJ709541 |
| *Labrus mixtus* | 5 |  | JQ775044; KJ768240-KJ768243 |
| *Symphodus tinca* |  | 1^◊^ | KJ709644 |
| *Xyrichtys novacula* |  | 3^◊^ | KJ709677-KJ709679 |
| **Family Ammodytidae** |  |  |  |
| *Hyperoplus lanceolatus* | 5 |  | JQ775039-  JQ775043 |
| **Family Trachinidae** |  |  |  |
| *Echiichthys vipera* | 5 |  | JQ775010-  JQ775014 |
| *Trachinus draco* | 6 | 6 | JQ774754;  JQ774928-  JQ774932; KJ709931-KJ709936 |
| *Trachinus radiatus* |  | 3 | KJ709937-KJ709939 |
| **Family Uranoscopidae** |  |  |  |
| *Uranoscopus scaber* † □ | 1 | 2 (1^◊^) | KJ709950; KJ709673; KJ768320 |
| **Family Blenniidae** |  |  |  |
| *Blennius ocellaris* | 7 | 8 (3^◊^) | JQ774786-  JQ774790;  JQ774983; KJ709703-KJ709707; KJ709487-KJ709489; KJ768218 |
| **Family Gobiesocidae** |  |  |  |
| *Lepadogaster lepadogaster* † | 3 |  | KJ768244-KJ768246 |
| **Family Callionymidae** |  |  |  |
| *Callionymus lyra* | 7 |  | JQ774580-  JQ774582;  JQ774794-  JQ774796;  JQ774988 |
| *Callionymus maculatus* | 1 |  | JQ774797 |
| *Synchiropus phaeton* | 4 | 1^◊^ | JQ774556;  JQ774749-  JQ774751; KJ709645 |
| **Family Gobiidae** |  |  |  |
| *Gobius niger* † | 1 |  | KJ768236 |
| *Pomatochistus lozanoi* | 5 |  | JQ775029-  JQ775033 |
| *Pomatoschistus marmoratus* |  | 2^◊^ | KJ709583-KJ709584 |
| *Pomatoschistus microps* † | 3 |  | KJ768285-KJ768287 |
| *Pomatoschistus tortonesei********* |  | 2^◊^ | KJ709585-KJ709586 |
| **Family Scombrolabrachidae** |  |  |  |
| *Scombrolabrax heterolepis* † | 1 |  | KJ768303 |
| **Family Sphyraenidae** |  |  |  |
| *Sphyraena sphyraena* |  | 2^◊^ | KJ709637-KJ709638 |
| **Family Gempylidae** |  |  |  |
| *Lepidocybium flavobrunneum* † | 1 |  | KJ768247 |
| **Family Trichiuridae** |  |  |  |
| *Benthodesmus simonyi* | 2 |  | JQ774573-  JQ774574 |
| *Lepidopus caudatus* † | 4 | 8 (4^◊^) | KJ709789-KJ709792; KJ709542-KJ709545; KJ768248-KJ768251 |
| **Family Scombridae** |  |  |  |
| *Auxis rochei rochei* † □ | 1 | 1^◊^ | KJ709483; KJ768211 |
| *Euthynnus alletteratus* |  | 1^◊^ | KJ709529 |
| *Sarda sarda* † | 4 | 1^◊^ | KJ709601; KJ768292-KJ768295 |
| *Scomber colias* † | 15 | 8 (3^◊^) | JQ774713-  JQ774719;  JQ775108-  JQ775114; KJ709873-KJ709877; KJ709606-KJ709608; KJ768298 |
| *Scomber scombrus* | 18 | 1^◊^ | JQ774720-  JQ774725;  JQ774901-  JQ774905;  JQ775115-  JQ775118; KJ709609; KJ768300-KJ768302 |
| *Thunnus alalunga* |  | 1^◊^ | KJ709651 |
| *Thunnus thynnus* |  | 1 | KJ709930 |
| **Family Xiphiidae** |  |  |  |
| *Xiphias gladius* |  | 2 (1^◊^) | KJ709951; KJ709676 |
| **Family Istiophoridae** |  |  |  |
| *Tetrapturus belone********* |  | 1 | KJ709929 |
| **Family Centrolophidae** |  |  |  |
| *Centrolophus niger* |  | 1 | KJ709721 |
| *Schedophilus medusophagus* |  | 1^◊^ | KJ709605 |
| **Family Caproidae** |  |  |  |
| *Antigonia capros* † | 1 |  | KJ768205 |
| *Capros aper* | 16 | 7 (2^◊^) | JQ774583-  JQ774588;  JQ774798-  JQ774799;  JQ774800-  JQ774802;  JQ774989-  JQ774993; KJ709713-KJ709717; KJ709493-KJ709494 |
| **Family Citharidae** |  |  |  |
| *Citharus linguatula* | 7 | 7 (2^◊^) | JQ774600-JQ774602;  JQ774813-  JQ774816; KJ709734-KJ709738; KJ709500-KJ709501 |
| **Family Scophthalmidae** |  |  |  |
| *Lepidorhombus boscii* | 9 | 2 (1^◊^) | JQ774842-  JQ774846;  JQ775045-  JQ775048; KJ709793; KJ709546 |
| *Lepidorhombus whiffiagonis* | 2 | 1 | JQ774536;  JQ774847; KJ709794 |
| *Scophthalmus rhombus* † | 1 |  | KJ768304 |
| **Family Pleuronectidae** |  |  |  |
| *Platichthys flesus* | 4 |  | JQ775094-  JQ775096; KJ768280 |
| **Family Bothidae** |  |  |  |
| *Arnoglossus imperialis* | 2 |  | JQ774774-  JQ774775 |
| *Arnoglossus laterna* | 9 | 4 | JQ774776-  JQ774780;  JQ774965-  JQ774968;  KJ709694-KJ709697 |
| *Arnoglossus rueppelii* | 10 |  | JQ774781-  JQ774785;  JQ774969-  JQ774973 |
| *Arnoglossus thori* |  | 5 | KJ709698-KJ709702 |
| *Bothus podas* |  | 1^◊^ | KJ709492 |
| **Family Soleidae** |  |  |  |
| *Bathysolea profundicola* † | 3 |  | KJ768213-KJ768215 |
| *Dicologlossa cuneata* | 1 |  | JQ775008 |
| *Microchirus azevia* | 5 |  | JQ774669-  JQ774672; KJ768257 |
| *Microchirus boscanion* | 5 |  | JQ774673-  JQ774677 |
| *Microchirus ocellatus* † | 1 | 1^◊^ | KJ709561; KJ768258 |
| *Microchirus variegatus* | 13 |  | JQ774678-  JQ774681;  JQ774864-  JQ774866;  JQ775076-  JQ775080; KJ768259 |
| *Pegusa impar* |  | 1^◊^ | KJ709575 |
| *Pegusa lascaris* † | 1 |  | KJ768276 |
| *Solea aegyptiaca********* |  | 1^◊^ | KJ709633 |
| *Solea senegalensis* | 2 |  | JQ775127; KJ768310 |
| *Solea solea* | 5 |  | JQ774918-  JQ774922 |
| **Family Cynoglossidae** |  |  |  |
| *Symphurus nigrescens* | 4 |  | JQ774552-  JQ774555 |
| **Family Balistidae** |  |  |  |
| *Balistes capriscus* † | 1 | 3^◊^ | KJ709484-KJ709486; KJ768212 |
| **Family Tetraodontidae** |  |  |  |
| *Sphoeroides pachygaster* † | 1 | 6 (1^◊^) | KJ709914-KJ709918; KJ709636; KJ768311 |
| **Family Molidae** |  |  |  |
| *Mola mola* | 2 |  | JQ775087-  JQ775088 |
| *Ranzania laevis* † | 1 |  | KJ768291 |
|  |  |  |  |
|  |  |  |  |

The number of specimens analyzed in each area is shown. NEA: North East Atlantic; MED: Mediterranean Sea. † identifies species newly analyzed in the reference dataset, in respect to Costa *et al.* [16]. □ new addition to the global COI-5P library. * Species with distribution limited to the Archipelago of the Azores and to the Strait of Messina. ** Species occurring only in the Mediterranean Sea ([www.fishbase.org](http://www.fishbase.org/)). ^◊^ identifies the number of specimens collected from commercial fisheries.
